# Empirical support for sequential imprinting during downstream migration in Atlantic salmon (*Salmo salar*) smolts

**DOI:** 10.1038/s41598-022-17690-2

**Published:** 2022-08-12

**Authors:** Tormod Haraldstad, Torbjørn Forseth, Esben M. Olsen, Thrond O. Haugen, Erik Höglund

**Affiliations:** 1 Laboratory of Freshwater Ecology and Inland Fisheries (LFI), NORCE Norwegian Research Centre AS, Universitetsveien 19, 4630 Kristiansand, Norway; 2 Norwegian Institute for Water Research, Grimstad, Norway; 3 Norwegian Institute for Nature Research, Trondheim, Norway; 4 Institute of Marine Research, Flødevigen, His Norway; 5 Department of Ecology and Natural Resource Management, Norwegian University of Life Sciences, Ås, Norway

**Keywords:** Animal migration, Behavioural ecology, Conservation biology

## Abstract

The precise homing of Atlantic salmon to their natal river and spawning grounds is the foundation for locally adapted genetically differentiated populations across rivers or across river sections. A sequential imprinting hypothesis states that salmon smolts may imprint on environmental clues along the outward migration route and then use this in reverse order to direct the spawning migration later in life. In this study, we provide empirical support for this hypothesis. PIT-tagged wild Atlantic salmon using a 2 km hydropower tunnel as downstream migrating smolts had a 18% (1SW) and 23% (2SW) lower probability of successfully migrating through the parallel river stretch as adult spawners compared to spawners that migrated through the same river stretch as smolts. These findings highlight how a fine-scale riverine migration route may be imprinted in wild Atlantic salmon smolts. From an applied perspective, these results stress the importance of not depriving smolts from parts of their migration route to ensure successful return of adults to their natal spawning grounds.

## Introduction

Migrations at ontogenetic shifts enable individuals to utilize the best suited habitat during different stages in their life cycle, and thus represents a strategy with potentially high adaptive value^1^. If migration is completed successfully, it can be associated with large growth- and fitness benefits^2^. However, on the one hand, such migration often comes with costs, including energy expenditures and elevated mortality risk.

The terms philopatry and homing refer to cases where individuals locate and reproduce in their nursery habitat^3^. It is a common fitness-related trait in many migratory animals, that increases likelihood of finding mates and locating habitats that are favorable for both adult reproduction and juvenile survival^4^. In particular, salmonids are known for their long-distance migrations at sea and precise homing to spawning and nursery habitats in rivers and streams^5^. This precise homing is associated with locally adapted genetically differentiated populations across rivers and even across sections within rivers^6^.

Anadromous salmonids seem to have a general sense of direction in the ocean, guiding them towards the coastal zone^7,8^. Navigation through the open sea may include the use of bi-coordinate map or compass systems^9,10^. When entering the coastal zone, salmonids may still navigate using these same mechanisms, however, visual and olfactory orientation seems to be increasingly important as the fish approaches coastal areas^11^.

It was previously hypothesized that juvenile salmonids imprint site specific environmental clues at their natal site and used these clues during homing^12,13^. Following this, tagging studies have revealed that salmonids return to the place they were released as smolts and not to their nursery river of genetic origin^14,15^, suggesting that clues obtained during smolt downstream migration are critical for precise return migration as spawners. Unlike smolts, adults are unable to learn the route back to a new river when transplanted as post-spawners^16^. Accordingly, physiological and neurological changes during the parr-smolt transformation have been linked to elevated olfactory sensitivity in salmonids^17,18^ and the existence of a sensitive period for olfactory imprinting has been demonstrated to occur 21–28 days after the onset of smoltification in Atlantic salmon^19^. During the smolt run, imprinting a series of consecutive events along the seaward migration route has been hypothesized to provide waypoints that can be recognized in reverse sequence during adult return migration^13^. Still, field-data from wild Atlantic salmon supporting this hypothesis is lacking^8^. Nevertheless, transport studies of hatchery reared salmon have shown that straying increased with increasing release distance from fresh water for both Atlantic salmon^20,21^ and pacific salmon^22,23^. In addition, pacific salmon transported around part of their smolt migration route in the Columbia River show higher straying rates than in-river migrants^24,25^. These migratory patterns suggest that increased straying rate in transported fish can be related to missed or disrupted imprinting events.

Here, we specifically evaluate to what extent smolt migration through hydropower tunnels might decrease homing efficiency by imprinting the turbine tunnel as an upstream migratory route and/or depriving fish from imprinting waypoints to the parallel river stretch that must be ascended by adults to reach upstream spawning grounds. To this end, we compared the upstream spawning migration of wild Atlantic salmon spawners PIT-tagged during smolt migration in spring 1 or 2 years earlier. During smolt migration, one group of smolts voluntarily migrated through a 2 km underground hydropower water tunnel and a turbine while the others voluntarily used a fish passage through the hydropower dam and into the 2.7 km minimum flow stretch (original riverbed) to the outlet of the hydropower water tunnel. Because adults must migrate through the minimum flow stretch, we hypothesised that the tunnel migrants had a lower probability of successfully ascending through this section as returning spawners, compared to river migrants.

## Methods

**Figure 1:**
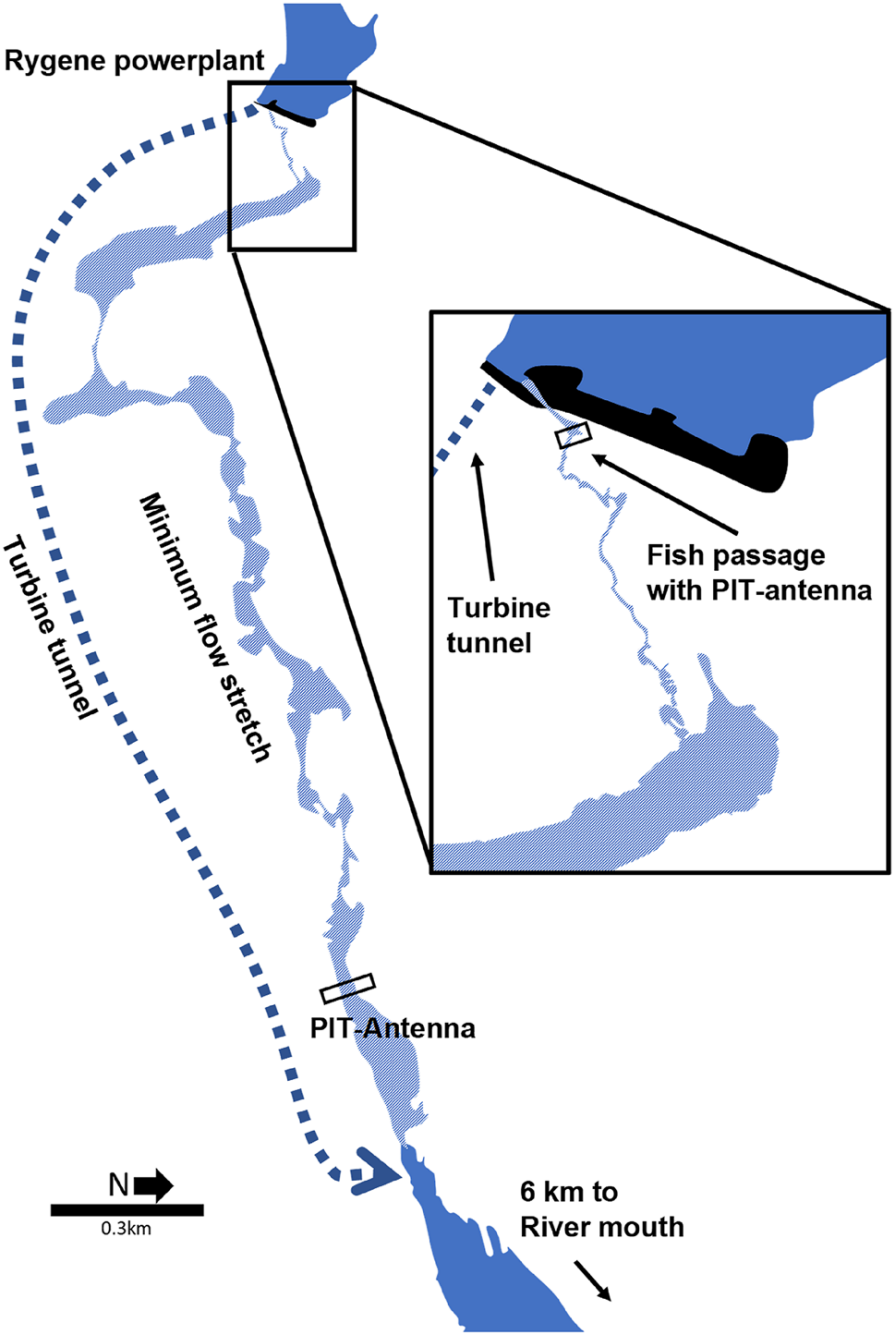
River Nidelva with Rygene hydropower dam with water intake and fish passage including the downstream minimum flow stretch with the parallel turbine water tunnel that exits into River Nidelva 6 km upstream of the river mouth. PIT-antennas in both ends of the minimum flow stretch detect returning PIT-tagged Atlantic salmon spawners.

The study was carried out in the River Nidelva, southern Norway (58.41540° N, 8.74242° E). The river lost its native Atlantic salmon population due to heavy acidification in the mid-1900s and a new population was established following liming^26^. The river has a mean annual discharge of 110 m^3^s^−1^ and Atlantic salmon use the lowermost 35 km as spawning and nursery habitat. Nidelva catchment is extensively regulated for hydropower both in the mountain areas and in the main river. The lowermost run-of-the-river hydropower plant has a dam and water intake at Rygene, 9 km upstream the river mouth (Fig. 1). From the dam forebay, the bulk of water is fed into a 2 km underground mountain tunnel with one Kaplan turbine and returned to the river, 6.4 km upstream the river mouth. This leaves a parallel 2.7 km minimum flow stretch in the old riverbed between the dam and water tunnel tailrace. The statutory water discharge is 1 m^3^s^−1^ in winter and 5 m^3^s^−1^ in fish migration periods in spring, summer and autumn.

During the smolt migration in spring, water is released through a surface fish passage in the dam and into the minimum flow stretch to aid the smolts past the turbine intake^27^. The fish passage is located perpendicular to the approaching flow on the eastern side of the submerged intake trash rack. The bar spacing in the trash rack is 80 mm, and thus large enough for smolts to pass. When in the hydropower forebay, the smolts are thus faced with a choice of two different migration alternatives; (1) through the trash rack and into the turbine tunnel or (2) through the surface fish passage and into the residual flow stretch. From the downstream junction between the two migration routes, Nidelva is slow flowing with increasing tidal influence towards the river mouth.

Upstream spawners have to ascend the minimum discharge stretch and a fish ladder through the hydropower dam at Rygene to reach the best spawning grounds. Thorstad et al.^28^ documented that upstream spawners spend several weeks at the turbine tunnel tail race, struggled to find the minimum flow stretch and few reached the fish ladder at Rygene dam. Since then, several weirs have been removed and an electric barrier has been established at the hydropower tunnel outlet. The number of salmon passing Rygene has increased over the last decades and migration peaks occurs earlier in the season^29^.

### Fish sampling and tagging

Downstream migrating wild Atlantic salmon smolts were caught in a Wolf-trap^30^ located in the entrance of the surface fish passage at Rygene hydropower plant. The smolts were anesthetized with MS222 (Metomidate, 2 mg/l) before being tagged internally with passive integrated transponder (PIT) tags (23 × 3.4 mm, 0.6 g in air, half duplex, Oregon RFID). The tag was inserted through a 3–4 mm incision made ventrally between the posterior tip of the pectoral fin and the anterior point of the pelvic girdle. Atlantic salmon smolts were tagged in 2014 (n = 536), 2016 (n = 1266) and 2017 (n = 2434). Permission to catch Atlantic salmon smolt in River Nidelva was granted by the County Governor of Agder. PIT-tagging was approved by Norwegian Animal Research Authority, NARA (FOTS ID 15463). All personnel involved in the tagging and handling of fish were trained and familiar with FELASA (Federal of European Laboratory Animal Science Associations) guidelines.

PIT-tagged smolts were released 100 m upstream of the hydropower dam where they could either migrate into the turbine tunnel or through the surface passage and into the minimum flow stretch. Individuals migrating through the fish passage were re-captured and identified using a handheld PIT-reader. Turbine migrants were not detected due to methodological limitation of PIT-antenna size and placement in such high-discharge/high-velocity tail-race area. Non-recaptured smolts were therefore assumed to be turbine migrants (29.5%). A recent study of acoustically tagged salmon smolts in Nidelva revealed high survival for smolts migrating through the turbine tunnel at Rygene (> 90%)^31^.

After the marine migration, returning PIT-tagged adult Atlantic salmon were registered by PIT-antennas during the 2016–2019 spawning runs to Nidelva. The overall return rates were 12.4%. The lowermost PIT-antenna located at the downstream end of the minimum flow stretch was a flatbed system that only covered half of the river width, thus only detecting a part of the returning PIT-tagged spawners. We assume random detection of tagged individuals and the analysis was only based upon returning adults that were detected on this lowermost antenna (n = 199). At Rygene dam a swim-through PIT-antenna loop was installed at the upper end of the minimum flow stretch, in the fish ladder that intersects the hydropower dam. Due to the narrow passage and reduced water volume, Atlantic salmon spawners were forced to pass close to the antenna covering the narrow opening between two consecutive pools in the fish ladder. Detection ranges were tested regularly with a 23 mm PIT tag and identified to cover the opening the salmon had to pass with additional ~ 0.3 m ranges on either side of the antenna plane. Antennas were wired to a remote tuner board and connected to an antenna reader box (TIRIS RI-CTL MB2A; Oregon RFID, USA) and supplied with 12 V power. When a tagged fish passed through the antenna loop, tag number, date and time were recorded and logged by the reader box.

### Statistical analysis

The statistical software R was used for data inspection and statistical analyses^32^. Three research questions were tested to investigate potential drivers for arrival time to the river (Arrival time model), probability of ascending the minimum flow stretch (Ascend success model) and the time spent in the minimum flow stretch (Progression time model). For each research question, candidate models were fitted with different combination of response variables. Model selection was based on Akaike’s information criterion^33,34^. We present the most supported model and comment on additional models that attained ΔAICc < 2. All model candidates attaining higher AICc support than 1% (i.e., AICc-weight) are provided in Supplementary Information. Residual plots of the selected models were studied for validation of assumptions of residual linearity and homoscedacity.

The arrival time data were analyzed using linear models (LM), fitted to evaluate potential drivers of arrival time (DoY = Day of Year) to river Nidelva. Candidate models included smolt migration route (hydropower tunnel or river), winters spent at sea before returning to spawn (1SW or 2SW) and smolt length (mm) as predictor variables. All combination of variables were tested, including interactions.

The probability of ascending through the residual flow stretch was addressed using generalized linear models. Successful ascendence were defined as detection in the uppermost PIT-antenna conditional on positive detection(s) in the lower antenna. The logit link function was used for linearization of the binomial response (0 = not sighted; 1 = sighted in uppermost PIT-antenna). Candidate models included smolt migration route (hydropower tunnel or river), arrival time to Nidelva (DoY registered in the lowermost PIT-antenna), winters spent at sea before returning to spawn (1SW or 2SW) and smolt length (mm) as predictor variables. All combination of variables were tested including interactions.

The progression time data were analyzed using linear models (LM), to address potential drivers of time used to ascend the minimum flow stretch. Only fish that had successful ascended the stretch were used in this analysis (n = 145, distance antenna A−B = 2.3 km). To account for the skewed progression time data, the response variable was log transformed. Candidate models included smolt migration experience (hydropower tunnel or river), arrival time to Nidelva (DoY registered in the lowermost PIT-antenna), winters spent at sea before returning to spawn (1SW or 2SW) and smolt length (mm) as predictor variables. All different combination of variables were tested including interactions.

## Results

Wild Atlantic salmon were tagged as smolts (mean length 151.7 ± 36.4 mm) at Rygene hydropower plant in river Nidelva. A fraction of the PIT-tagged returning spawners was detected in the PIT-antenna after spending one (1SW, n = 149) or two (2SW, n = 50) winters at sea. In total, 52 salmon had migrated through the turbine tunnel as smolts while 147 migrated through the minimum flow stretch. Returning Atlantic salmon spawners entered River Nidelva between 3 June (DoY = 154) and 6 November (DoY = 310) in the years 2016–2019.

Model selection favored an effect of the number of winters spend at sea (SW) and smolt length (mm) on the arrival time to Nidelva (Table 1). The best model had a 56% support (Table S1). The second-best model (AICc = 1.88) included an additive effect of migration route as smolts in addition to winters spend at sea and smolt length (22% support). The selected model predicted that salmon that had spent 2 years at sea (2SW) entered the river 20 days prior to the salmon that had spent 1 year at sea (1SW) and that spawners that were large as smolts entered the river earlier in the season (e.g., spawners with a smolt length at 180 mm enter the river 26 days earlier than a spawner that were 130 mm as smolt).

**Table 1:** Logit-parameter estimates and corresponding likelihood-ratio test statistics for the most supported model fitted to predict arrival time (DOY = Day of Year) to River Nidelva for Atlantic salmon spawners PIT tagged as smolts (smolt length) that had spendt one (1SW) or two (2SW) winters at sea before returning to spawn in River Nidelva.

Parameter estimates			LR-test statistics			
Term	Coeff	SE	Effect	d*f*	*χ*^2^	*p*
Intercept	295.57	26.95	Sea winter (2SW)	1	20344.9	< 0.001
Sea winter (2SW)	− 20.42	5.10	Smolt length (mm)	1	8188.2	0.003
Smolt length (mm)	− 0.53	0.18				

**Figure 2:**
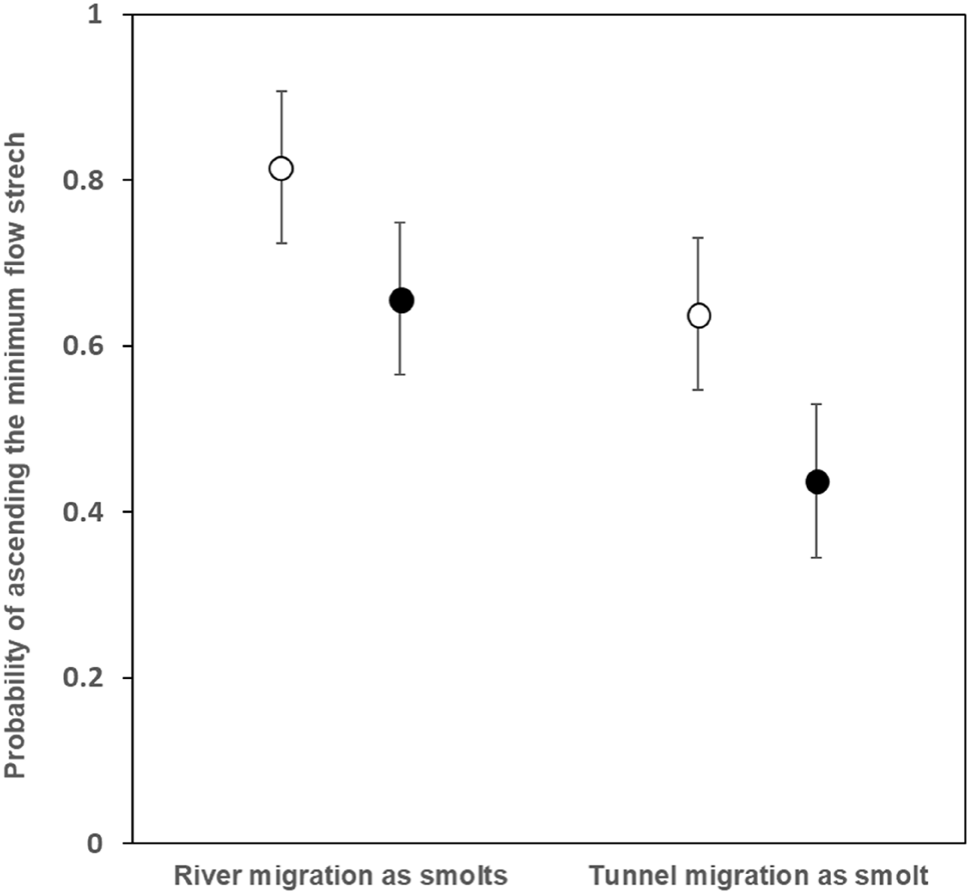
Predicted Atlantic salmon spawners probability to ascend through the minimum flow stretch in River Nidelva for individual river and tunnel migrants as smolts as a function of number of years at sea before returning to spawn (1SW:white points, 2SW black points, with standard error) to River Nidelva derived from the selected binomial GLM reported in Table 2.

Of the 199 spawners that were detected in the lowermost PIT-antenna, 72.9% (n = 145) were registered 2.3 km upstream in the uppermost antenna located in the fish ladder at Rygene dam. Model selection supported an additive effect of migration route as smolts and sea age on the probability of ascending successfully through the minimum flow stretch. (Table 2, Fig. 2). This model attained 37% AICc support and an AICc‐score 1.19 lower than the second-most supported model that included smolt length in addition to migration experience as smolt and winter spent at sea (Table S2). All the five best models included smolt migration route as one of the predictors. The selected model predicted a 18% (1SW) and 23% (2SW) higher probability for successful ascendence through the minimum flow stretch for salmon spawners that had migrated through the minimum flow stretch as smolts compared to the salmon spawners that had used the hydropower tunnel as smolts. In addition, one sea winter (1SW) salmon had a higher probability (22% for turbine migrants as smolts; 17% for minimum flow stretch migrants as smolt) for successful migration through the minimum flow stretch than two sea winter (2SW) salmon.

**Table 2:** Logit-parameter estimates and corresponding likelihood-ratio test statistics for the most supported GLM model fitted to predict probabilities for Atlantic salmon spawners to ascend the minimum flow stretch for induviduals that migrated through two different routes as smolts (minimum flow stretch or hydropower tunnel) and spendt one (1SW) or two (2SW) winters at sea before returning to spawn in River Nidelva.

Parameter estimates			LR-test statistics			
Term	Coeff	SE	Effect	d*f*	*χ*^2^	*p*
Intercept	1.53	0.23	Smolt route (Tunnel)	1	7.77	0.008
Smolt route (Tunnel)	− 0.93	0.35	Sea winter (2SW)	1	6.21	0.012
Sea winter (2SW)	− 0.89	0.36

Atlantic salmon spawners used median 7.3 days (range 1.2–113.5 days, IQR = 11.0 days) to ascend the minimum flow stretch from the lowermost PIT-antenna to the PIT-antenna at Rygene dam. Model selection supported an effect of arrival day on progression time in the residual flow stretch (39% support, Table 3). Two other models attained ΔAIC < 2, both included arrival day and an interaction with smolt length (25% support) or additive effect (24% support) of smolt length (Table S3). All the five most supported models included arrival day as one of the predictor variables. The selected model predicted a decline from 12.1 days for spawners detected June 15 (166 DoY) to 4.3 days for spawners detected September 15 (258 DoY).

**Table 3:** Logit-parameter estimates and corresponding likelihood-ratio test statistics for the most supported model fitted to predict progression time through the minimum flow stretch for Atlantic salmon spawners arriving to the lowermost PIT-antenna in River Nidelva at different dates (DoY).

Parameter estimates			LR-test statistics			
Term	Coeff	SE	Effect	d*f*	*χ*^2^	*p*
Intercept	3.267	0.246	Arrival DoY	1	2.846	< 0.001
Arrival DoY	− 0.005	0.001				

## Discussion

This study found that Atlantic salmon using an artificial tunnel rather than a natural river section as smolts had a lower probability of ascending successfully through the river section as adult spawners. This finding provides empirical support for the sequential imprinting hypothesis stating that clues in the river were memorized sequentially during a few critical days as downstream migrating smolts and then used in reverse order as navigation clues for upstream migration spawners years later. The management implication is that whenever possible smolts should not be deprived from parts of their migration route to ensure successful return of adults to their natal spawning grounds.

This study strongly suggests that individuals with an incomplete imprint of river sections as smolts have a lower probability of successfully ascending deprived stretches as spawners. Several studies highlight how hatchery-reared and escaped farmed Atlantic salmon enter rivers later in the season and have a variable within-river migratory pattern compared to wild salmon^35–37^. In addition, experimental release of smolts in rivers, coastal zone and feeding areas at sea revealed that experienced gained as outward migrating smolts is required for Atlantic salmon to navigate precisely along the coast, into the fjords and towards the home river^7^. We acknowledge that the upstream spawning migration of Atlantic salmon is complex and effected by both intrinsic and environmental factors not addressed in this study^38^. However, the understanding of general mechanisms stimulating fish within-river migration are still lacking. The present study gives new insight into the river migration by showing that wild salmon deprived from a section of the river as smolts had a lower probability of ascending the section as adults. This finding lends support to the hypothesis of sequential imprinting of the smolt migration route^13^ and highlight how detailed the imprinted riverine migration route in wild Atlantic salmon smolts is.

Mitigation measures for anadromous salmonids at hydropower dams requires solutions that secure two-way migration with measures devoted to both downstream migrating smolts (e.g., fish bypasses) and upstream migrating spawners (e.g., fishways)^39^. Separate solutions for up- and downstream migration are essential for coping with the difference in behaviour, size and swimming performance between the two life stages (smolts vs. adult spawners). However, given the result from the present study showing how detailed smolts imprint their migration route indicates that success of upstream migration through fish ladders might be influenced by the experience of descending smolts. If smolts are aided too far downstream through tunnels or pipes, their ability to find the correct upstream migration route may be challenged and fewer adults may return to the upstream production areas. Rather, smolts should be guided past hydropower plant water intakes and released close to structures constructed to guide upstream spawners. This might increase the probability of individual imprint critical points which result in high passage success of measures that guide upstream migrating spawners.

In the present study, we show the delayed effects of depriving smolts from part of their migration route. Several measures have been made to increase smolt survival in regulated rivers, like developing more fish friendly turbines^40,41^, and the commonly used strategy of transporting migratory fish past river sections heavily changed by humans (trap and haul)^42^. These measures may enhance short-term smolt survival, however, the delayed effects may manifest in adult stages by increased straying rates. Studies of Pacific salmon from the Columbia river basin show that barge-transported juvenile chinook salmon (*Oncorhynchus tshawytscha*) and steelhead (*Oncorhynchus mykiss*) strayed more frequently than river migrants^43^. It should be noted, however, that when mortality in specific river stretch is high, the benefit of transport may outweigh the reduced local homing due to poor imprinting. The results call for a need to assess the lifetime fitness effects of various mitigation measures for smolts in hydropower regulated rivers. Cessation of such measures might increase smolt mortality, but at the same time secure individual imprint on critical points which result in higher total return rates to upstream spawning grounds and higher lifetime fitness.

We note that individuals best suited to find the fish passages as smolts might also be best at ascending hindrances in the minimum flow stretch as adults. A recent study by Haraldstad et al.^44^ document how individual differences in behavioural traits affect migration route choice for descending Atlantic salmon smolts at hydropower plants. If these individual behavioural differences persist during the smolt run and subsequent marine migration, they may be reflected in the observed difference in adult migration success between fish passage and turbine tunnel migrants. Potentially, individuals that hold traits associated with successfully passing man-made structures have tremendous fitness benefits in regulated rivers. The potential for hydropower to induced selection on such traits could be large on affected fish populations. We encourage further research on this field as it has attained minimum attention so far^45^.

Atlantic salmon that spent two winters at sea had a lower probability of ascending the minimum flow stretch than one sea winter fish. These findings are supported by Jonsson et al.^46^ who found that the straying rates from River Imsa increased with time spent at sea. If salmonid smolts are capable of memorizing sequential clues it is also possible that the longer they stay at sea some of this information gets lost due to memory decay, as seen in other fish species^47^. The ultimate consequences from this pattern of higher probability of straying in individuals that mature later at larger sizes remain enigmatic, and ought to be subject to future studies. Addressing the combined effects from smolt migration route and sea-age at maturity on life-time reproduction success are of particular interest as deprivation of access to natal spawning areas may pose a considerable fitness cost that may disfavor late-maturing genotypes.

In conclusion, this study supports the hypothesis that cues in the river is memorized sequentially by Atlantic salmon during a few critical days as downstream migrating smolts, which are then used as navigation for upstream migrating adult spawners later in life. This finding stresses the importance of carefully considering mitigation measures that involves depriving smolts from parts of their migration route as it decreases the likelihood of returning to their natal spawning grounds, essential to maintain locally adapted and viable populations.

## Supplementary Information

Supplementary Tables.
