# Supplementary material for: Empirical support for sequential imprinting during downstream migration in Atlantic salmon (Salmo salar) smolts
Source: Sci Rep. 2022 Aug 12;12:13736. doi: 10.1038/s41598-022-17690-2 (PMC9374756; doi:10.1038/s41598-022-17690-2)
Supplement: Supplementary file 1 — Supplementary Tables. [file 41598_2022_17690_MOESM1_ESM.docx]

## Supplementary Information

**Table S1**. Model selection table for the most supported candidate LM-models fitted to predicted arrival time of tagged Atlantic salmon at the lowermost PIT-antenna in River Nidelva on their spawning migration. All models attained a support larger than 0.01% are included. K = number of parameters fitted, AICc = corrected Akaike’s information criterion, ΔAICc = difference between a model’s AICc value and the most supported model, AICcWt = the relative AICc support of the model, *LL*=the model log likelihood. Route= Downstream migration route as smolts one to two years prior to spawning migration (0=Minimum flow stretch; 1=Hydropower tunnel). SW= Number of years at sea before returning to spawn (1SW; 2SW). S-length= Total length in mm when tagged as smolts one to two years prior to spawning.

| Model structure | K | AICc | ΔAICc | AICcWt | Cum.Wt | *LL* |
| --- | --- | --- | --- | --- | --- | --- |
| SW + S-length | 4 | 1932.04 | 0 | 0.56 | 0.56 | -961.92 |
| Route + SW + S-length | 5 | 1933.93 | 1.88 | 0.22 | 0.77 | -961.81 |
| SW * S-length | 5 | 1934.15 | 2.10 | 0.19 | 0.97 | -961.92 |
| SW | 3 | 1938.62 | 6.58 | 0.02 | 0.99 | -966.25 |
| Route + SW | 4 | 1940.37 | 8.32 | 0.01 | 1.00 | -966.08 |

**Table S2**. Model selection table for the most supported candidate GLM-models fitted to predict the probability of ascending through the minimum flow stretch. Successful ascendence were defined as detection in the uppermost PIT-antenna conditional on positive detection in the lower antenna. All models attained a support larger than 0.01% are included. K = number of parameters fitted, AICc = corrected Akaike’s information criterion, ΔAICc = difference between a model’s AICc value and the most supported model, AICcWt = the relative AICc support of the model, *LL*=the model log likelihood. Route= Downstream migration route as smolts one to two years prior to spawning migration (0=Minimum flow stretch; 1=Hydropower tunnel). SW= Number of years at sea before returning to spawn (1SW; 2SW). S-length= Total length in mm when tagged as smolts one to two years prior to spawning. Arrival= Arrival time to River Nidelva (DoY registered in the lowermost PIT-antenna).

| Model structure | K | AICc | ΔAICc | AICcWt | Cum.Wt | *LL* |
| --- | --- | --- | --- | --- | --- | --- |
| Route + SW | 3 | 224.81 | 0 | 0.37 | 0.37 | -109.34 |
| Route + S-length + SW | 4 | 226.00 | 1.19 | 0.20 | 0.57 | -108.90 |
| Route * SW | 4 | 226.87 | 2.06 | 0.13 | 0.70 | -109.33 |
| Route * S-length * Arrival | 8 | 228.79 | 3.97 | 0.05 | 0.75 | -106.01 |
| Route | 2 | 228.96 | 4.15 | 0.05 | 0.80 | -112.45 |
| Arrival | 3 | 229.34 | 4.53 | 0.04 | 0.84 | -111.61 |
| S-length * Arrival | 4 | 229.36 | 4.55 | 0.04 | 0.88 | -110.58 |
| SW | 2 | 229.66 | 4.85 | 0.03 | 0.91 | -112.80 |
| Route + S-length | 3 | 230.82 | 6.01 | 0.02 | 0.93 | -112.35 |
| S-length + SW | 3 | 231.02 | 6.21 | 0.02 | 0.94 | -112.45 |
| Route * Arrival | 4 | 231.38 | 6.57 | 0.01 | 0.96 | -111.59 |
| Route + S-length + Arrival | 4 | 231.40 | 6.59 | 0.01 | 0.97 | -111.60 |
| Route * S-length * SW | 8 | 231.90 | 7.09 | 0.01 | 0.98 | -107.57 |
| S-length * SW | 4 | 232.41 | 7.60 | 0.01 | 0.99 | -112.10 |
| Route * S-length | 4 | 232.90 | 8.09 | 0.01 | 1.00 | -112.35 |

**Table S3**. Model selection table for the most supported candidate LM-models fitted to predict potential drivers of time used to ascend the minimum flow stretch. All models attained a support larger than 0.01% are included. K = number of parameters fitted, AICc = corrected Akaike’s information criterion, ΔAICc = difference between a model’s AICc value and the most supported model, AICcWt = the relative AICc support of the model, *LL*=the model log likelihood. Route= Downstream migration route as smolts one to two years prior to spawning migration (0=Minimum flow stretch; 1=Hydropower tunnel). SW= Number of years at sea before returning to spawn (1SW; 2SW). S-length= Total length in mm when tagged as smolts one to two years prior to spawning. Arrival= Arrival time to River Nidelva (DoY registered in the lowermost PIT-antenna).

| Model structure | K | AICc | ΔAICc | AICcWt | Cum.Wt | *LL* |
| --- | --- | --- | --- | --- | --- | --- |
| Arrival | 3 | 154.07 | 0 | 0.39 | 0.39 | -73.95 |
| S-length * Arrival | 5 | 154.94 | 0.87 | 0.25 | 0.64 | -72.25 |
| S-length + Arrival | 4 | 155.05 | 0.98 | 0.24 | 0.88 | -73.38 |
| Route + S-length + Arrival | 5 | 157.09 | 3.01 | 0.09 | 0.97 | -73.33 |
| Route * S-length + Arrival | 6 | 159.26 | 5.19 | 0.03 | 1.00 | -73.32 |
